# Enhanced Cellular Uptake of Compact Cas Proteins: A Comparative Study of Cas12f and Cas9 in Human Cells

**DOI:** 10.1002/elsc.70042

**Published:** 2025-09-26

**Authors:** Karim E. Shalaby, Issam Hmila, S. M. Nasir Uddin, Nasser H. Zawia, Omar M. A. El‐Agnaf, Mustapha Aouida

**Affiliations:** ^1^ Laboratory of Pluripotent Stem Cell Disease Modeling, Translational Medicine Department Research Branch, Sidra Medicine Doha Qatar; ^2^ Neurological Disorder Research Center, Qatar Biomedical Research Institute (QBRI) Hamad Bin Khalifa University (HBKU), Qatar Foundation Doha Qatar; ^3^ Biological and Biomedical Sciences Division College of Health & Life Sciences Hamad Bin Khalifa University Doha Qatar; ^4^ Department of Biomedical and Pharmaceutical Sciences, College of Pharmacy University of Rhode Island Kingston Rhode Island USA; ^5^ George and Anne Ryan Institute for Neuroscience University of Rhode Island Kingston Rhode Island USA; ^6^ Interdisciplinary Neuroscience Program University of Rhode Island Kingston Rhode Island USA

**Keywords:** Cas12f, Cas9, CRISPR, delivery, ribonucleoprotein (RNP)

## Abstract

The clinical translation of CRISPR genome‐editing therapies is often hindered by inefficient delivery of the CRISPR‐Cas RNA‐protein complex into target cells. The most widely used CRISPR‐Cas9 system poses a significant challenge for efficient delivery into cells due to its large size (∼1.4 kDa). Recently reported compact Cas proteins, such as Cas12f (552 Da), Cas12k (639 Da), and Cas12m (596 Da) represent attractive alternatives as cargoes for delivery. In this brief research report, we employ efficient delivery vectors to evaluate the efficiency of cellular uptake of a compact Cas protein (Cas12f) compared to the widely used larger Cas9 in human cells. Our findings demonstrate that compact Cas proteins may facilitate more efficient cellular penetration and delivery, making them a promising alternative for the development of CRISPR‐based therapies.

Practical Application:

Our study demonstrates that compact Cas proteins significantly enhance cellular uptake compared to larger Cas proteins. This improved uptake efficiency suggests that compact Cas proteins could be more effective for clinical application, where size constraints and delivery efficiency are critical challenges. Combined with the optimization and refinement of the editing efficiencies of compact Cas systems, our study provokes further exploration of compact Cas proteins in various therapeutic contexts to advance the development of more efficient CRISPR‐based therapies.

**Abbreviations**
CRISPR: clustered regularly interspaced short palindromic repeats
PF14: PepFect14
RNP: ribonucleoprotein

## 1 Introduction

The CRISPR‐Cas genome‐editing system has emerged as a transformative technology for modifying genetic material with high precision [1, 2]. However, its clinical and research applications are limited by challenges in delivering the CRISPR ribonucleoprotein (RNP) complexes efficiently into target cells [3]. The large size of the most common Cas proteins such as SpCas9 with a size of 1.4 kDa, Cas12a (1.3 kDa), and Cas13 (1.1 kDa) can limit their delivery efficiency to cells. Miniature Cas proteins such as Cas12f (552 Da), Cas12k (639 Da), and Cas12m (596 Da) can overcome this limitation.

Amphipathic carriers are well established efficient delivery vectors of CRISPR RNPs to human cells for the purpose of genome editing [4, 5, 6, 7, 8, 9]. Like Cas9 RNPs, with a theoretical net charge of –80, Cas12f RNPs are also negatively charged (theoretical net charge: –202) and can form complexes with the cationic hydrophilic portion of amphipathic carriers, while the hydrophobic section facilitates endocytosis. In this brief report, we used amphipathic peptide as well as lipid‐based vectors to investigate whether delivering a more compact Cas protein, herein Cas12f, would result in increased levels of cellular uptake compared to the most commonly used Cas9. Our findings highlight the cellular uptake advantages of compact Cas proteins, demonstrating their potential for advancing genome‐editing applications.

## 2 Materials and Methods

### 2.1 Cell Lines and Culture

Human embryonic kidney (HEK293T) cells were obtained from ATCC and cultured in Dulbecco's Modified Eagle Medium (DMEM) (Thermo Fisher Scientific, USA) supplemented with 10% fetal bovine serum (FBS) (Sigma–Aldrich, USA) and 1% penicillin‐streptomycin (Thermo Fisher Scientific, USA). Cells were maintained at 37°C in a humidified atmosphere with 5% CO_2_.

### 2.2 CRISPR RNP Complex Formation

Recombinant Cas9 protein was purchased from New England Biolabs (NEB), USA. pET28a‐SUMO‐AsCas12f‐2NLS was a gift from Quanjiang Ji (Addgene plasmid # 171613) [10]. Cas12f was expressed in BL21(DE3) *Escherichia coli* and purified by the Ni‐nitrilotriacetic acid (NTA)‐based chromatography (Figure S1). Guide RNAs targeting a gene sequence were synthesized by Integrated DNA Technologies (IDT), USA, and are indicated in Table S1. The RNP complexes were prepared by incubating Cas9 or Cas12f proteins with sgRNA at a molar ratio of 1:1.1 in nuclease‐free duplex buffer (IDT, USA) at 25°C (Cas9) or 45°C (Cas12f) for 10 min.

### 2.3 RNP‐Peptide Complex Formation

PepFect14 (PF14) peptide was purchased from Pepscan, USA. Cas9/PF14 and Cas12f/PF14 complexes were formed by adding PF14 at the desired molar ratios to 6 *p*mol RNPs in a total of 100 µL HEPES‐buffered glucose solution (20 mM HEPES, 5% glucose, pH = 7.2) and vortexing immediately for 2 s. The mixture was incubated for 40 min at room temperature to form RNP/PF14 complexes.

### 2.4 Transmission Electron Microscopy (TEM)

Complexes prepared for transfection were applied directly to a TEM grid. Complexes containing 2.5 µg of Cas protein were deposited onto a carbon‐coated TEM grid (300 mesh) and incubated for 2 min. The grids were then fixed with 0.5% glutaraldehyde, rinsed with nuclease‐free distilled deionized water, and stained with 2% uranyl acetate for 1 min. After air drying, the grids were analyzed.

### 2.5 Transfection

HEK293T cells were seeded at a density of 15,000 cells per well in 100 µL FreeStyle293 medium (Thermo Fischer Scientific, USA) in a 96‐well plate 24 h before transfection. For peptide transfection, 20 µL of RNP/PF14 complexes containing 1.2 *p*mol RNP (RNP:PF14 molar ratio 1:100) were added to the cells. For transfection using lipofectamine, Lipofectamine CRISPRMAX (Thermo Fisher Scientific, USA) was used to transfect 1.2 *p*mol RNP, according to manufacturer's instructions. After 4 h, cells were topped up with 100 µL FreeStyle293 media containing 20% FBS.

### 2.6 MTT Assay

RNP/PF14 complexes (RNP:PF14 molar ratio 1:100) were formed as described above and added to cells at the indicated amounts. MTT reagent (Sigma–Aldrich, USA) was added 24 h after transfection and assay was carried out according to manufacturer's instructions. Absorbance was then measured at 570 nm using a plate reader.

### 2.7 Evaluation of Cellular Uptake

RNP complexes were prepared as mentioned above with a gRNA fluorescently labeled with ATTO^550^ (IDT, USA) before transfection. After 6 and 24 h, cells were analyzed using a fluorescence microscope, and uptake of the ATTO^550^ fluorescently‐labeled gRNA was quantified using a BD FACSAria flow cytometer using a PE‐CF594 filter. Results were expressed as percentages of uptake and mean fluorescence intensity ratios (MFIR) (ratio of treated cells/untreated control).

## 3 Results

### 3.1 Complex Formation of Cas12f and Cas9 RNPs With PF14

**FIGURE 1:**
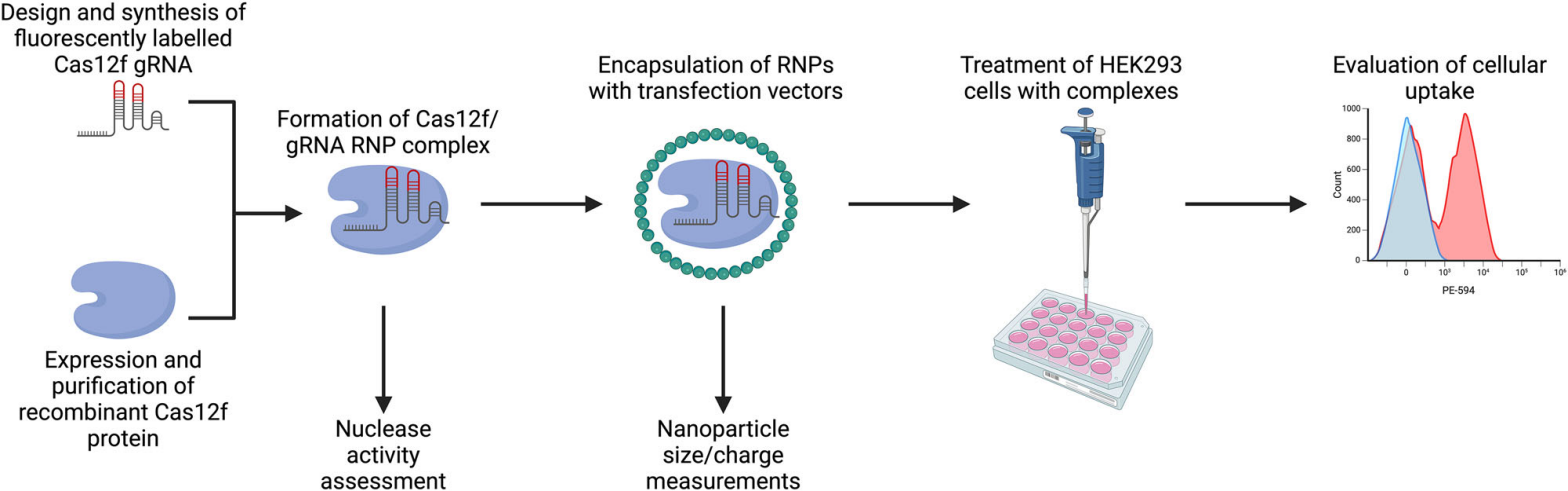
**Study workflow comparing the cellular uptake of Cas9 and Cas12f**. Recombinant Cas12f protein was expressed, purified, and complexed with a fluorescently labeled single guide RNA (sgRNA) targeting a known target. The nuclease activity of the assembled RNP complex was validated through an in vitro cleavage assay. Functional RNP complexes were encapsulated within transfection vectors (Lipofectamine or PF14) and characterized using dynamic light scattering (DLS) for particle size and zeta potential and transmission electron microscopy (TEM) for structural analysis. Cellular uptake was evaluated by treating cells with the complexes, followed by fluorescence microscopy and flow cytometry analysis using a PE‐CF594 filter. (Figure generated using BioRender.com).

Efficient delivery of Cas RNPs relies on vectors that ensure cellular internalization while preserving the functionality of the complexes. Non‐viral vectors, such as amphipathic peptides, have demonstrated potential as delivery agents due to their ability to form nanosized complexes and mediate cellular uptake through hydrophobic interactions. Among these, PepFect14 (PF14), an amphipathic peptide, has shown success in delivering Cas9 RNPs, achieving comparable efficiency to lipid‐based vectors like Lipofectamine [7, 8]. Through hydrophobic interactions, amphipathic peptides are not only capable of binding RNPs but also achieving levels of cellular internalization resembling lipid‐based transfection reagents. We used PF14 and Lipofectamine as efficient and different methods for delivery to compare the uptake of a smaller Cas protein, Cas12f, with the widely used larger Cas9 (Figure 1).

**FIGURE 2:**
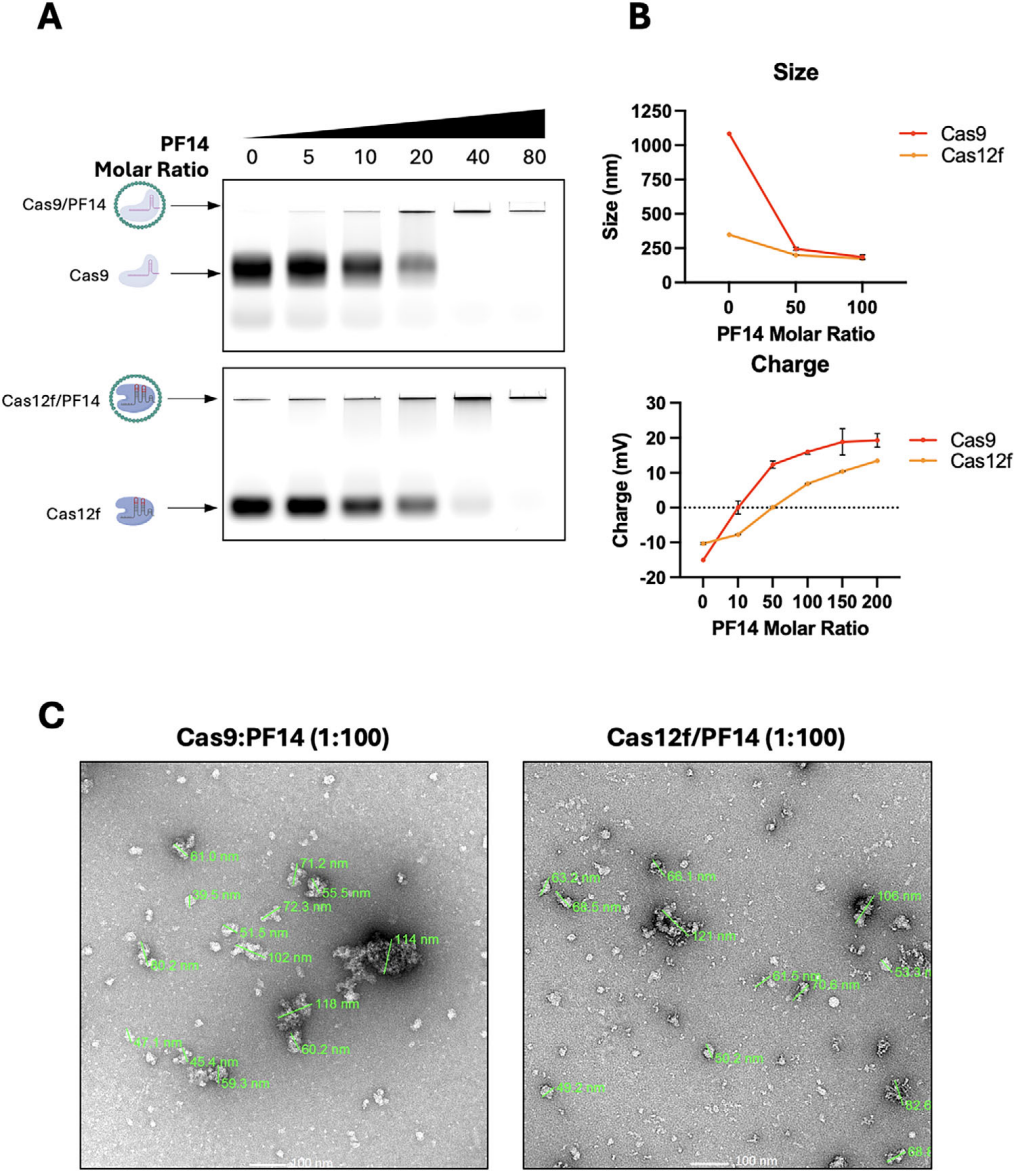
**Characterization of Cas9/PF14 and Cas12f/PF14 complexes. (A)** Gel‐shift assay showing the binding and complexing of PF14 peptide with Cas9 and Cas12f RNPs at increasing molar ratios. PF14 fully complexes Cas9 RNPs at a molar ratio of 40 and Cas12f RNPs at a molar ratio of 80, as indicated by retention of RNPs in the wells. **(B)** Dynamic light scattering (DLS) analysis of Cas9/PF14 and Cas12f/PF14 complexes showing smaller hydrodynamic diameters for Cas12f complexes (∼250 nm) compared to Cas9 complexes (∼1100 nm). Both complexes condensed to ∼150 to 200 nm with a positive charge at higher molar ratios (1:50 or above). (**C)** Transmission electron microscopy (TEM) images of Cas9/PF14 and Cas12f/PF14 particles at a 1:100 molar ratio, revealing particle diameters of 50–120 nm.

To test whether PF14 (net charge +5) could similarly bind and complex Cas12f RNPs and determine the optimal ratio for full complexing, PF14 was added to Cas12f RNPs at increasing molar ratios in a gel‐shift assay (Figure 2A). PF14 was able to bind and fully complex Cas9 RNPs at a molar ratio of 40, and Cas12f RNPs at a molar ratio of 80 as shown by the retention of RNPs to the wells of the gel (Figure 2A). The higher ratio required to fully neutralize the charge of Cas12f RNPs could be explained by the higher net negative charge (–202) carried by those RNPs compared with Cas9 RNPs (net charge –80).

To characterize and compare the biophysical properties of the Cas9/PF14 and Cas12f/PF14 complexes, particle size and zeta‐potential analyses were carried out (Figure 2B). Dynamic light scattering (DLS) showed that Cas12f RNPs, with a hydrodynamic diameter of ∼250 nm, were smaller in size compared to Cas9 RNPs (∼1100 nm). This is expected due to the smaller size of the Cas12f protein (∼51 kDa) compared to Cas9 (>150 kDa). At molar ratios 1:50 and above, Cas9/PF14 and Cas12f/PF14 complexes were condensed to a size of 150–200 nm and carried a positive charge (Figure 2B). Transmission electron microscopy (TEM) displayed the formation of Cas9/PF14 and Cas12f/PF14 particles of 50–120 nm in diameter at an RNP:PF14 molar ratio of 1:100 (Figures 2C and S2).

### 3.2 Improved Cellular Uptake of Cas12f RNP Complexes

**FIGURE 3:**
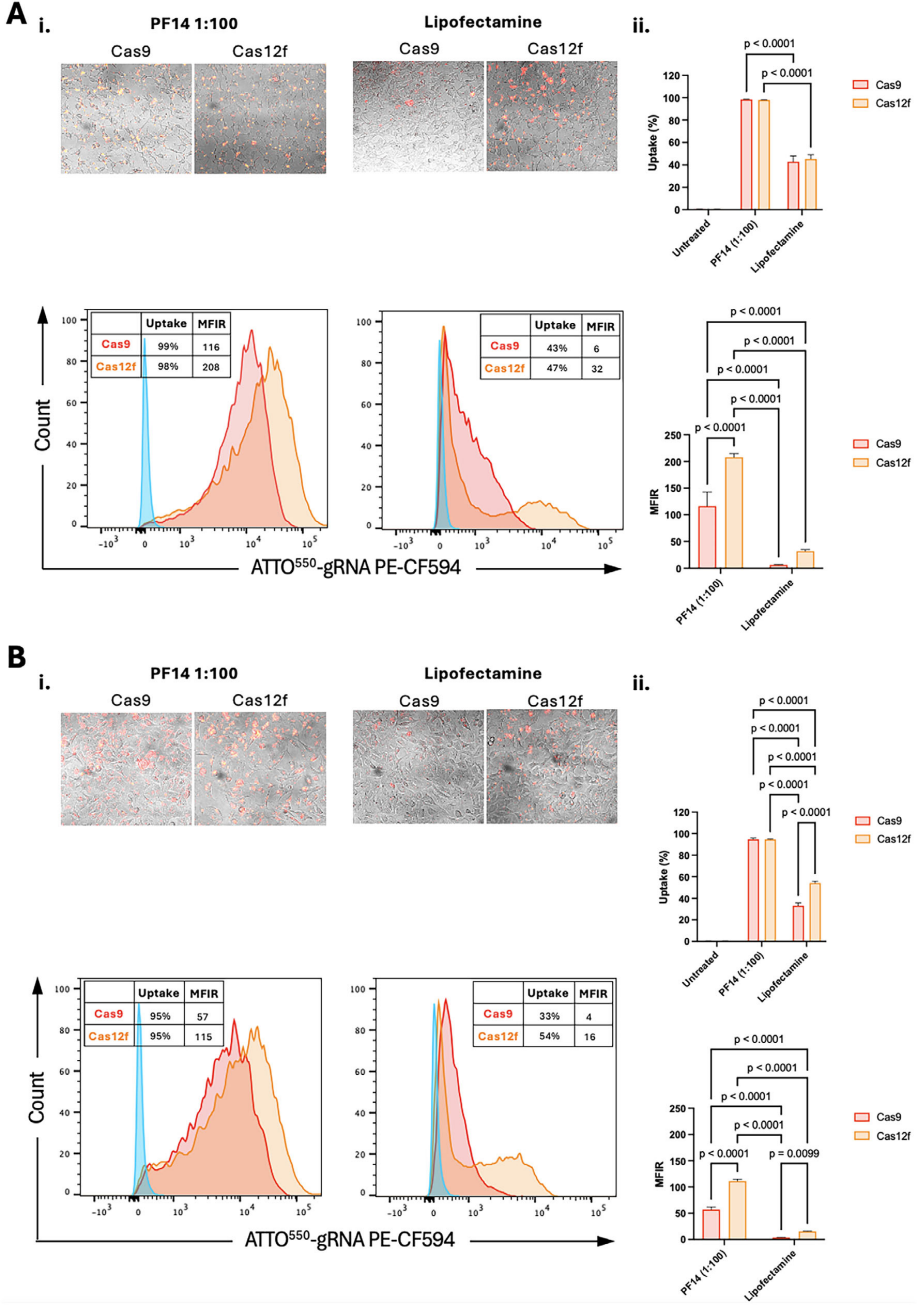
**Cellular uptake of Cas9 and Cas12f RNP complexes in HEK293 cells. (A)** (i) Representative fluorescence microscopy images and flow cytometry analysis at 6 h post‐transfection showing over 90% uptake for both Cas9 (red) and Cas12f (orange) RNPs when delivered with PF14, and less than 50% uptake using Lipofectamine CRISPRMAX. Treatments were compared to control untreated cells (blue). (ii) Mean fluorescence intensity ratio (MFIR) analysis reveals significantly higher molecular uptake for Cas12f RNPs compared to Cas9 RNPs, irrespective of delivery method (PF14 or Lipofectamine CRISPRMAX), indicating enhanced intracellular uptake facilitated by the smaller size of Cas12f RNPs (*n* = 3). Comparisons were made using two‐way ANOVA. The data are presented as mean ± SEM. (B) (i) Representative fluorescence microscopy images and flow cytometry analysis at 24 h post‐transfection. Uptake percentages remained above 90% for Cas9 (red) and Cas12f (orange) RNPs delivered by PF14. A significant increase in Cas12f RNP uptake was observed compared to Cas9 RNPs when using Lipofectamine. Treatments were compared to control untreated cells (blue). (ii) Mean fluorescence intensity ratio (MFIR) analysis reveals significantly higher molecular uptake for Cas12f RNPs compared to Cas9 RNPs, irrespective of delivery method (PF14 or Lipofectamine CRISPRMAX), indicating enhanced intracellular uptake facilitated by the smaller size of Cas12f RNPs (*n* = 3). Comparisons were made using two‐way ANOVA. The data are presented as mean ± SEM.

Cellular uptake events can start immediately after transfection [11, 12]. Thus, comparing the uptake efficiency of Cas9 and Cas12f is best done at the initial binding with cellular receptors, with the resolution decreasing as the receptors become saturated. We used flow cytometry to measure and compare the cellular uptake of equimolar amounts of Cas9 and Cas12f RNPs at 6 and 24 h post‐transfection in HEK293T cells. The Cas9 and Cas12f RNP/PF14 used were non‐toxic to cells as was shown by MTT assay (Figure S3). At 6 h, the percentage of cells that uptook Cas9 or Cas12f RNPs did not vary and represented ∼100% of cells with PF14 peptide transfection, and ∼45% with Lipofectamine transfection (Figure 3A). At 24 h, the percentages of cells with RNP uptake remained above 90% with PF14, and decreased only for Cas9% to 33% but remained at similar levels for Cas12f with Lipofectamine, indicating sustained presence of Cas12f in cells (Figure 3B).

At both 6 and 24 h, mean fluorescence intensity ratio (MFIR) analysis revealed significantly higher levels of RNP uptake in cells transfected with Cas12f compared to Cas9 using either PF14 or Lipofectamine, suggesting enhanced molar uptake of the smaller Cas12f protein (Figure 3Aii,Bii). At 24 h, MFIRs were reduced by ∼50%, indicating protein clearance, with Cas12f remaining at significantly higher levels than Cas9. This observation highlights the potential advantages of smaller sized cargoes in enhancing cellular uptake and improving the overall efficiency of CRISPR delivery systems.

## 4 Discussion

Compact Cas proteins carry promise for enhanced delivery for CRISPR‐based therapies. Significantly higher levels of RNP uptake were achieved in Cas12f transfected cells compared with cells transfected with the larger Cas9 protein (Figure 3Aii,3Bii). This indicates that a significantly higher number of RNP uptake events occurred per cell which may be relevant to CRISPR‐based therapies, where efficient delivery is necessary.

Here, we carried out particle size measurements of RNP‐peptide complexes (Figure 2B). PF14 peptide effectively reduced the size of both Cas9 and Cas12f cargoes to similarly sized complexes (<200 nm) for efficient cell penetration (Figure 2B,C). Although Cas9/PF14 and Cas12f/PF14 complexes formed particles of the same size, these particles likely carry nonequimolar amounts of RNPs due to the inherent size difference between Cas9 (∼1.4 kDa) and Cas12f (∼0.4 kDa) proteins. Thus, a particle of Cas12f/PF14 likely engulfs a significantly higher number of RNP molecules than does a particle of Cas9/PF14, which is reflected by the consistent higher fluorescence intensity detected in cells transfected with Cas12f compared with cells transfected with Cas9 (Figure 3Aii,3Bii).

The findings here underscore the potential of compact Cas proteins as more efficient alternatives to the more widely used larger Cas proteins such as Cas9 and Cas12a, particularly for therapeutic delivery where size constraints and delivery efficiency are critical challenges. Research exploring novel compact Cas analogs or enhancing their gene‐editing efficiency could be synergistic with their enhanced delivery efficiency to develop alternatives to larger Cas proteins. Such advances would likely result in more robust therapeutic outcomes and broaden the applicability of CRISPR‐based therapies.

This brief research report provides preliminary data for further exploring compact Cas proteins applicability for therapeutic applications. Further work is required to compare the nuclease activity of different sized Cas analogs, optimized for efficiency, in relevant disease models. Additionally, optimizing the formulation of delivery vectors such as ligands for cell‐specific delivery will be crucial for advancing CRISPR‐based therapies toward clinical use.

## Ethics Statement

Not applicable.

## Consent

All authors gave their consent for publication.

## Conflicts of Interest

The authors declared no conflicts of interest.

## Supporting information




**Supporting File 1:** elsc70042‐sup‐0001‐SuppMat.docx

## Data Availability

The datasets used and/or analyzed during the current study are included in the manuscript.
