# Supplementary material for: Enhanced Cellular Uptake of Compact Cas Proteins: A Comparative Study of Cas12f and Cas9 in Human Cells
Source: Eng Life Sci. 2025 Sep 26;25(9):e70042. doi: 10.1002/elsc.70042 (PMC12464724; doi:10.1002/elsc.70042)
Supplement: Supplementary file 1 — Supporting File 1: elsc70042‐sup‐0001‐SuppMat.docx [file ELSC-25-e70042-s001.docx]

**Supplementary Information**

Enhanced Cellular Uptake of Compact Cas: A Comparative Study of Cas12f and Cas9 in Human Cells

Karim E. Shalaby^1,3^, Issam Hmila^3^, S. M. Nasir Uddin^2^, Nasser H. Zawia^3,4,5,6^, Omar M. A. El-Agnaf^3^, Mustapha Aouida^2†^

1: Laboratory of Pluripotent Stem Cell Disease Modeling, Translational Medicine Department, Research Branch, Sidra Medicine, Doha, Qatar

2: Biological and Biomedical Sciences Division, College of Health & Life Sciences, Hamad Bin Khalifa University, Doha, Qatar

3: Neurological Disorder Research Center, Qatar Biomedical Research Institute (QBRI), Hamad Bin Khalifa University (HBKU), Qatar Foundation, Doha, Qatar

4: Department of Biomedical and Pharmaceutical Sciences, College of Pharmacy, University of Rhode Island, Kingston, RI 02881, USA

5: George and Anne Ryan Institute for Neuroscience, University of Rhode Island, Kingston, RI 02881, USA

6: Interdisciplinary Neuroscience Program, University of Rhode Island, Kingston, RI 02881, USA

†Correspondence: Mustapha Aouida ([maouida@hbku.edu.qa](mailto:maouida@hbku.edu.qa))

**Table of Content:**

1. **Supplementary Figure 1: Cas12f purification.**
2. **Supplementary Figure 2. Representative transmission electron microscopy images of blank (buffer), PF14, Cas9 RNP, and Cas12f RNP.**
3. **Supplementary figure 3. Cytotoxicity tests for Cas9 and Cas12f RNP/PF14 using MTT assay.**
4. **Table 1: Guide RNAs (gRNA) used for forming ribonucleoprotein complexes.**
5. **DNA plasmid used for in-vitro cleavage.**

**
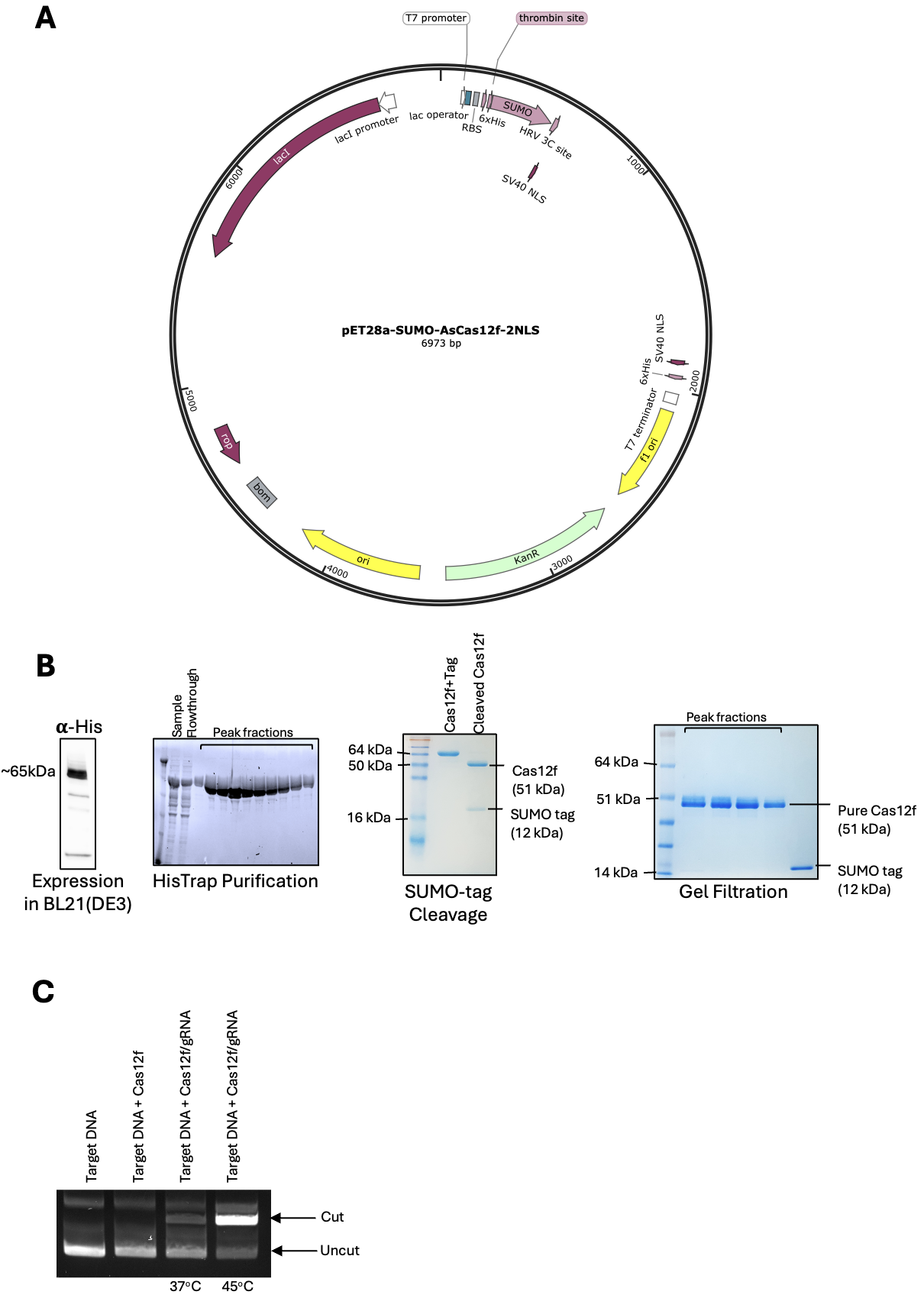
Supplementary Figure 1:** **Cas12f purification.** (A) Plasmid map of the Cas12f purification construct, designed for expression in BL21(DE3) *E. coli*. (B) Overview of the Cas12f expression and purification process. Following IPTG induction of BL21(DE3) *E. coli* cells harboring the Cas12f plasmid, lysates were subjected to nickel-affinity chromatography, yielding a highly purified Cas12f protein preparation, as verified by SDS-PAGE. (C) In vitro cleavage assay demonstrating the DNA-editing activity of purified Cas12f. A target DNA substrate was cleaved efficiently, confirming the functional integrity and nuclease activity of the purified Cas12f protein

**Supplementary Figure 2.** **Representative transmission electron microscopy images of blank (buffer), PF14, Cas9 RNP, and Cas12f RNP.**


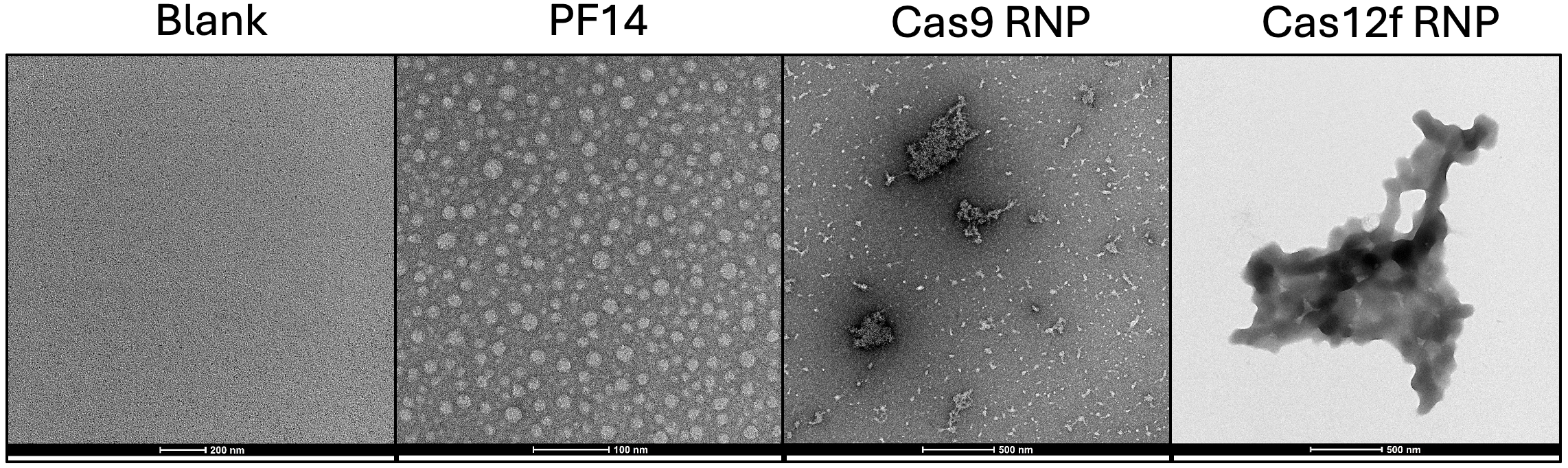


**Supplementary figure 3. Cytotoxicity tests for Cas9 and Cas12f RNP/PF14 using MTT assay.** HEK293T cells were transfected with increasing amounts of Cas9/PF14 or Cas12f/PF14 complexes and MTT assay was carried out after 24 hours.


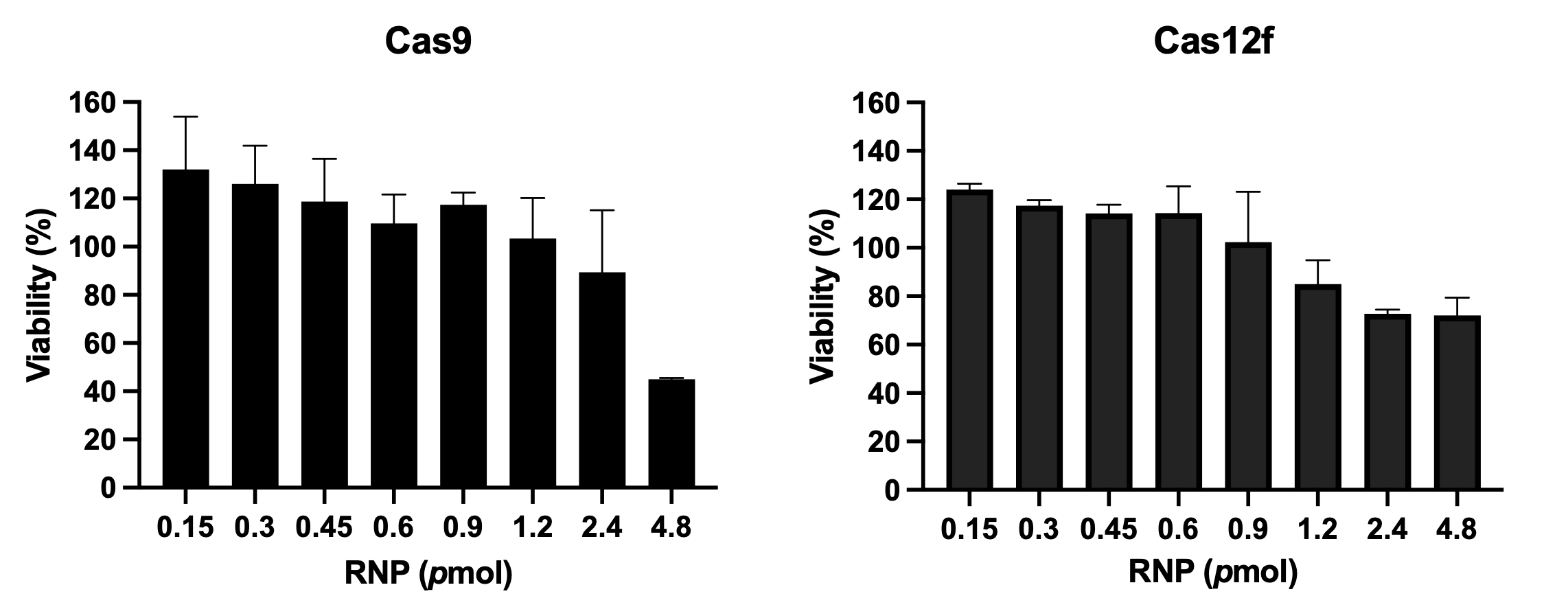


**Table 1: Guide RNAs (gRNA) used for forming ribonucleoprotein complexes:**

| gRNA | Sequence (crRNA target underlined) |
| --- | --- |
| Cas9 | 5’-CUGCUGUGACUGCUUGUAGAGTTTTAGAGCTAGAAATAGCAAGTTAAAATAAGGCTAGTCCGTTATCAACTTGAAAAAGTGGCACCGAGTCGGTGC-3’ |
| Cas12f | 5’-GGGAUUCGUCGGUUCAGCGACGAUAAGCCGAGAAGUGCCAAUAAAAGUGGUUUGGUAACGCUCGGUAAGGUAGCCAAAAGGCUGAAACUCCGUGCACAAAGACCGCACGGACGCUUCACAUAUAGCUUGUGGAGUGUGAACUAGAUGGCCAUGGCGCGGAC -3’ |

**DNA plasmid used for in-vitro cleavage (target Sequence in yellow, cloned between BamHI and BstXI):**

tagttattaatagtaatcaattacggggtcattagttcatagcccatatatggagttccgcgttacataacttacggtaaatggcccgcctggctgaccgcccaacgacccccgcccattgacgtcaataatgacgtatgttcccatagtaacgccaatagggactttccattgacgtcaatgggtggagtatttacggtaaactgcccacttggcagtacatcaagtgtatcatatgccaagtacgccccctattgacgtcaatgacggtaaatggcccgcctggcattatgcccagtacatgaccttatgggactttcctacttggcagtacatctacgtattagtcatcgctattaccatggtgatgcggttttggcagtacatcaatgggcgtggatagcggtttgactcacggggatttccaagtctccaccccattgacgtcaatgggagtttgttttggcaccaaaatcaacgggactttccaaaatgtcgtaacaactccgccccattgacgcaaatgggcggtaggcgtgtacggtgggaggtctatataagcagagctggtttagtgaaccgtcagatccgctagccaccatggcctcctccgaggacgtcatcaaggagttcatgcgcttcaaggtgcgcatggagggctccgtgaacggccacgagttcgagatcgagggcgagggcgagggccgcccctacgagggcacccagaccgccaagctgaaggtgaccaagggcggccccctgcccttcgcctgggacatcctgtcccctcagttccagtacggctccaaggcctacgtgaagcaccccgccgacatccccgactacttgaagctgtccttccccgagggcttcaagtgggagcgcgtgatgaacttcgaggacggcggcgtggtgaccgtgacccaggactcctccctgcaggacggcgagttcatctacaaggtgaagctgcgcggcaccaacttcccctccgacggccccgtaatgcagaagaagaccatgggctgggaggcctccaccgagcggatgtaccccgaggacggcgccctgaagggcgagatcaagatgaggctgaagctgaaggacggcggccactacgacgccgaggtcaagaccacctacatggccaagaagcccgtgcagctgcccggcgcctacaagaccgacatcaagctggacatcacctcccacaacgaggactacaccatcgtggaacagtacgagcgcgccgagggccgccactccaccggcgccgaattcGCGTCCGCGCCATGGCCATCTACAAGCAGTCACAGCAggatccagtgagcaagggcgaggagctgttcaccggggtggtgcccatcctggtcgagctggacggcgacgtaaacggccacaagttcagcgtgtccggcgagggcgagggcgatgccacctacggcaagctgaccctgaagttcatctgcaccaccggcaagctgcccgtgccctggcccaccctcgtgaccaccctgacctacggcgtgcagtgcttcagccgctaccccgaccacatgaagcagcacgacttcttcaagtccgccatgcccgaaggctacgtccaggagcgcaccatcttcttcaaggacgacggcaactacaagacccgcgccgaggtgaagttcgagggcgacaccctggtgaaccgcatcgagctgaagggcatcgacttcaaggaggacggcaacatcctggggcacaagctggagtacaactacaacagccacaacgtctatatcatggccgacaagcagaagaacggcatcaaggtgaacttcaagatccgccacaacatcgaggacggcagcgtgcagctcgccgaccactaccagcagaacacccccatcggcgacggccccgtgctgctgcccgacaaccactacctgagcacccagtccgccctgagcaaagaccccaacgagaagcgcgatcacatggtcctgctggagttcgtgaccgccgccgggatcactctcggcatggacgagctgtacaagtaaagcggccgccagtgagcaagggcgaggagctgttcaccggggtggtgcccatcctggtcgagctggacggcgacgtaaacggccacaagttcagcgtgtccggcgagggcgagggcgatgccacctacggcaagctgaccctgaagttcatctgcaccaccggcaagctgcccgtgccctggcccaccctcgtgaccaccctgacctacggcgtgcagtgcttcagccgctaccccgaccacatgaagcagcacgacttcttcaagtccgccatgcccgaaggctacgtccaggagcgcaccatcttcttcaaggacgacggcaactacaagacccgcgccgaggtgaagttcgagggcgacaccctggtgaaccgcatcgagctgaagggcatcgacttcaaggaggacggcaacatcctggggcacaagctggagtacaactacaacagccacaacgtctatatcatggccgacaagcagaagaacggcatcaaggtgaacttcaagatccgccacaacatcgaggacggcagcgtgcagctcgccgaccactaccagcagaacacccccatcggcgacggccccgtgctgctgcccgacaaccactacctgagcacccagtccgccctgagcaaagaccccaacgagaagcgcgatcacatggtcctgctggagttcgtgaccgccgccgggatcactctcggcatggacgagctgtacaagtaagcggccgcgactctagatcataatcagccataccacatttgtagaggttttacttgctttaaaaaacctcccacacctccccctgaacctgaaacataaaatgaatgcaattgttgttgttaacttgtttattgcagcttataatggttacaaataaagcaatagcatcacaaatttcacaaataaagcatttttttcactgcattctagttgtggtttgtccaaactcatcaatgtatcttaaggcgtaaattgtaagcgttaatattttgttaaaattcgcgttaaatttttgttaaatcagctcattttttaaccaataggccgaaatcggcaaaatcccttataaatcaaaagaatagaccgagatagggttgagtgttgttccagtttggaacaagagtccactattaaagaacgtggactccaacgtcaaagggcgaaaaaccgtctatcagggcgatggcccactacgtgaaccatcaccctaatcaagttttttggggtcgaggtgccgtaaagcactaaatcggaaccctaaagggagcccccgatttagagcttgacggggaaagccggcgaacgtggcgagaaaggaagggaagaaagcgaaaggagcgggcgctagggcgctggcaagtgtagcggtcacgctgcgcgtaaccaccacacccgccgcgcttaatgcgccgctacagggcgcgtcaggtggcacttttcggggaaatgtgcgcggaacccctatttgtttatttttctaaatacattcaaatatgtatccgctcatgagacaataaccctgataaatgcttcaataatattgaaaaaggaagagtcctgaggcggaaagaaccagctgtggaatgtgtgtcagttagggtgtggaaagtccccaggctccccagcaggcagaagtatgcaaagcatgcatctcaattagtcagcaaccaggtgtggaaagtccccaggctccccagcaggcagaagtatgcaaagcatgcatctcaattagtcagcaaccatagtcccgcccctaactccgcccatcccgcccctaactccgcccagttccgcccattctccgccccatggctgactaattttttttatttatgcagaggccgaggccgcctcggcctctgagctattccagaagtagtgaggaggcttttttggaggcctaggcttttgcaaagatcgatcaagagacaggatgaggatcgtttcgcatgattgaacaagatggattgcacgcaggttctccggccgcttgggtggagaggctattcggctatgactgggcacaacagacaatcggctgctctgatgccgccgtgttccggctgtcagcgcaggggcgcccggttctttttgtcaagaccgacctgtccggtgccctgaatgaactgcaagacgaggcagcgcggctatcgtggctggccacgacgggcgttccttgcgcagctgtgctcgacgttgtcactgaagcgggaagggactggctgctattgggcgaagtgccggggcaggatctcctgtcatctcaccttgctcctgccgagaaagtatccatcatggctgatgcaatgcggcggctgcatacgcttgatccggctacctgcccattcgaccaccaagcgaaacatcgcatcgagcgagcacgtactcggatggaagccggtcttgtcgatcaggatgatctggacgaagagcatcaggggctcgcgccagccgaactgttcgccaggctcaaggcgagcatgcccgacggcgaggatctcgtcgtgacccatggcgatgcctgcttgccgaatatcatggtggaaaatggccgcttttctggattcatcgactgtggccggctgggtgtggcggaccgctatcaggacatagcgttggctacccgtgatattgctgaagagcttggcggcgaatgggctgaccgcttcctcgtgctttacggtatcgccgctcccgattcgcagcgcatcgccttctatcgccttcttgacgagttcttctgagcgggactctggggttcgaaatgaccgaccaagcgacgcccaacctgccatcacgagatttcgattccaccgccgccttctatgaaaggttgggcttcggaatcgttttccgggacgccggctggatgatcctccagcgcggggatctcatgctggagttcttcgcccaccctagggggaggctaactgaaacacggaaggagacaataccggaaggaacccgcgctatgacggcaataaaaagacagaataaaacgcacggtgttgggtcgtttgttcataaacgcggggttcggtcccagggctggcactctgtcgataccccaccgagaccccattggggccaatacgcccgcgtttcttccttttccccaccccaccccccaagttcgggtgaaggcccagggctcgcagccaacgtcggggcggcaggccctgccatagcctcaggttactcatatatactttagattgatttaaaacttcatttttaatttaaaaggatctaggtgaagatcctttttgataatctcatgaccaaaatcccttaacgtgagttttcgttccactgagcgtcagaccccgtagaaaagatcaaaggatcttcttgagatcctttttttctgcgcgtaatctgctgcttgcaaacaaaaaaaccaccgctaccagcggtggtttgtttgccggatcaagagctaccaactctttttccgaaggtaactggcttcagcagagcgcagataccaaatactgtccttctagtgtagccgtagttaggccaccacttcaagaactctgtagcaccgcctacatacctcgctctgctaatcctgttaccagtggctgctgccagtggcgataagtcgtgtcttaccgggttggactcaagacgatagttaccggataaggcgcagcggtcgggctgaacggggggttcgtgcacacagcccagcttggagcgaacgacctacaccgaactgagatacctacagcgtgagctatgagaaagcgccacgcttcccgaagggagaaaggcggacaggtatccggtaagcggcagggtcggaacaggagagcgcacgagggagcttccagggggaaacgcctggtatctttatagtcctgtcgggtttcgccacctctgacttgagcgtcgatttttgtgatgctcgtcaggggggcggagcctatggaaaaacgccagcaacgcggcctttttacggttcctggccttttgctggccttttgctcacatgttctttcctgcgttatcccctgattctgtggataaccgtattaccgccatgcat
